# Development and psychometric evaluation of a physician global assessment for type 2 systemic lupus erythematosus symptoms

**DOI:** 10.1136/lupus-2023-001016

**Published:** 2023-12-17

**Authors:** Megan E B Clowse, Jennifer L Rogers, Theresa Coles, David S Pisetsky, Lisa G Criscione-Schreiber, Dana Burshell, Jayanth Doss, Rebecca E Sadun, Kai Sun, Mithu Maheswaranathan, Amanda M Eudy

**Affiliations:** 1 Division of Rheumatology and Immunology, Duke University School of Medicine, Durham, North Carolina, USA; 2 Population Health Sciences, Duke University School of Medicine, Durham, North Carolina, USA; 3 Durham VA Medical Center, Durham, North Carolina, USA

**Keywords:** Outcome Assessment, Health Care, Lupus Erythematosus, Systemic, Lupus Nephritis

## Abstract

**Objective:**

Manifestations of SLE can be categorised as type 1 (classic signs and symptoms of SLE) or type 2 (fatigue, widespread pain and brain fog with an unclear relationship to inflammation). While measures of type 1 SLE activity exist, most current physician-reported measures do not encompass type 2 SLE manifestations. To better evaluate type 2 SLE symptoms, we developed and psychometrically evaluated a physician-reported measure of type 2 symptoms, the Type 2 Physician Global Assessment (‘Type 2 PGA’).

**Methods and analysis:**

The Type 2 PGA was developed and evaluated by six rheumatologists practising in the same academic lupus clinic. The study began with a roundtable discussion to establish consensus guidelines for scoring the Type 2 PGA. Following the roundtable, the Type 2 PGA was psychometrically evaluated using data prospectively collected from 263 patients with SLE enrolled in the Duke Lupus Registry.

**Results:**

There was strong intra-rater and inter-rater reliability (intraclass correlation coefficient=0.83), indicating the Type 2 PGA scores were consistent within a rheumatologist and across rheumatologists. The Type 2 PGA was correlated with patient-reported symptoms of polysymptomatic distress (r=0.76), fatigue (r=0.68), cognitive dysfunction (r=0.63), waking unrefreshed (r=0.62) and forgetfulness (r=0.60), and weakly correlated with the Type 1 PGA and the Systemic Lupus Erythematosus Disease Activity Index.

**Conclusion:**

The Type 2 PGA performed well as a physician-reported measure of type 2 SLE symptoms. The incorporation of the Type 2 PGA into a routine rheumatology visit may improve patient care by bringing the provider’s attention to certain symptoms not well represented in conventional measures of disease activity.

**What is already known on this topic**

- Type 2 SLE symptoms of fatigue, widespread pain and brain fog are frequently reported as major symptoms by people living with SLE.
- Most physician-reported measures do not measure the severity of type 2 SLE symptoms.

**What this study adds**

- We developed and validated a novel physician-reported measure of type 2 symptoms, the Type 2 Physician Global Assessment (Type 2 PGA).
- The Type 2 PGA had high intra- and inter-rater reliability, and an increase in polysymptomatic distress correlated with an increase in Type 2 PGA score.

**How this study might affect research, practice or policy**

- The Type 2 PGA may be an efficient and reliable way to measure type 2 SLE activity over time, providing a useful tool for both clinical care and research.
- Analogous to the commonly used SLE physician/provider global assessments, the Type 2 PGA is an additional measure to evaluate changes in patient symptoms, correlate with physiological changes and quantify response to treatment.

## Introduction

While some manifestations and symptoms of SLE are closely related to inflammation, other symptoms have an uncertain relationship to inflammation despite their prominence and severity. In 2019, we proposed a new model for assessing the full range of symptoms experienced by patients with SLE: the Type 1 and 2 SLE Model.^1^ In this model, type 1 SLE manifestations include signs and symptoms that physicians traditionally associate with active inflammatory SLE, including arthritis, rash and proteinuria. Type 2 SLE manifestations include symptoms frequently reported as major concerns by people living with SLE but are largely not represented in physician scores; such symptoms include widespread pain, fatigue, depression and brain fog. Type 2 SLE symptoms have a broad array of causes, ranging from lupus-related inflammation to insomnia, all of which can be exacerbated by stress, disease perception and catastrophising. The Type 1 and 2 SLE Model is a working conceptual model that continues to be iteratively revised based on patient and provider feedback, as well as evolving data analysis.

While the immunological mechanisms driving type 1 symptoms have been extensively investigated and targeted with therapies, the basis of type 2 symptoms is largely unknown. Type 2 symptoms are, by definition, largely subjective and patient-reported, with an as-yet undetermined relationship to the pathophysiology of inflammatory SLE, which complicates the approach to therapy. In our lupus clinic and research, type 2 symptoms have primarily been measured using the Polysymptomatic Distress Scale (PSD), derived from the 2011/2016 American College of Rheumatology (ACR) fibromyalgia criteria.^2–4^

Because type 2 symptoms contribute greatly to the reduced quality of life experienced by people with SLE, it is our goal to understand the aetiology of these symptoms. To do so, we must be able to reproducibly measure these symptoms. Therefore, analogous to physician/provider global assessments (PGAs) used in research across rheumatic illnesses to document providers’ estimate of inflammatory disease activity, we began developing a provider-reported measure of type 2 symptoms. A validated type 2 PGA will serve two purposes: (1) enable research into type 2 lupus symptoms, their correlates and responses to therapy; and (2) improve patient care by acknowledging that the lived experience of lupus activity has multiple dimensions. Assessing and addressing both dimensions during a clinic visit will improve the delivery of patient-centred care.

Therefore, we created and began exploring a provider-reported measure of type 2 symptoms, the Type 2 Physician Global Assessment (Type 2 PGA). Similar 4-point scales comprise the Lupus Foundation of America-Rapid Evaluation of Activity in Lupus (LFA-REAL) and the Lupus Activity Index (LAI) for the assessment of SLE activity and are included in the Safety of Estrogens in Lupus National Assessment-Systemic Lupus Erythematosus Disease Activity Index (SELENA-SLEDAI) Flare Index.^5–7^ We started using the Type 2 PGA within the note template of our practice in 2018 to determine the feasibility of integrating it into routine clinical care. The goal of the current project was to evaluate the psychometric properties of the Type 2 PGA.

## Materials and methods

### Patient population

The Duke Lupus Clinic meets weekly and is staffed by six attending rheumatologists. All Duke Lupus Clinic patients meeting the 1997 ACR or Systemic Lupus International Collaborating Clinics criteria for SLE are invited to enrol in the Duke Lupus Registry (DLR).^8 9^ Patients enrolled in the DLR are ≥18 years old and signed an informed consent to participate.

### Data collection

Data have been collected in the DLR since 2018. At each clinic visit, patients completed questionnaires, including the PSD and the Systemic Lupus Activity Questionnaire (SLAQ).^2–4 10 11^ The PSD consists of two subscales: the Widespread Pain Index (WPI) and the Symptom Severity Score (SSS).^2–4^ The WPI asks patients to indicate in which of 19 areas of the body they experienced pain in the prior month. For the SSS, patients reported the extent of their fatigue, cognitive symptoms and waking unrefreshed over the past month. In addition, patients reported if they had headache, pain or cramps in the lower abdomen or depression in the last 6 months. The total PSD score is a composite of the WPI and SSS, ranging from 0 to 31; higher scores indicate more polysymptomatic distress. The PSD score is classified into severity categories: none (0–3), mild (4–7), moderate (8–11), severe (12–19) and very severe (20–31).^3^

The SLAQ is a 24-symptom patient-reported measure of SLE activity in the past month.^10 11^ Within the SLAQ, patients reported if they had a flare and rated their lupus disease activity on a scale from 0 (no activity) to 10 (most activity). Patients also reported their experiences of a range of symptoms, rating the severity of each symptom as mild, moderate or severe.

Our treating rheumatologists completed disease activity measures at each clinic visit, including the Systemic Lupus Erythematosus Disease Activity Index (SLEDAI), Type 1 PGA and Type 2 PGA.^6 12 13^ We began collecting the Type 2 PGA in the Duke Lupus Clinic note in 2018 as part of a quality improvement project to increase management of type 2 SLE symptoms. Laboratory values and medications were recorded at each visit. All data were stored in a REDCap database.

### Psychometric data collection

#### Step 1: roundtable discussion

The Type 2 PGA is scored on a severity scale of 0–3. Prior to the psychometric evaluation of the Type 2 PGA, the six rheumatologists in the Duke Lupus Clinic, who had already been recording Type 2 PGAs for approximately 2 years, came to a consensus on the level of type 2 SLE symptoms for which they would typically or frequently introduce a treatment. The participating rheumatologists also discussed the symptom components of type 2 SLE to consider when scoring the Type 2 PGA. Roundtable procedures were derived from bookmarking studies.^14^

##### Case review

Before the roundtable, the providers independently scored the Type 2 PGA for a panel of 30 patient cases selected from the DLR who were seen in 2018–2019. The sample cases were chosen to represent a range of type 2 severity as determined by the PSD, type 1 SLE activity determined by the SLEDAI and patient demographics. For each de-identified case, the providers were given (1) patient-completed questionnaires for the visit being scored (including the SLAQ and the PSD); (2) the clinical note for the visit being scored, including medications; and (3) the clinical note from the prior visit. Type 1 and Type 2 PGA scores were removed from selected clinic notes prior to review; the reviewers did not know patients’ race or ethnicity. The providers scored each case’s Type 2 PGA on a scale from 0 (no activity) to 3 (most severe activity) in 0.25 intervals. During these discussions, we identified ‘guideposts’ for scoring the Type 2 PGA. A guidepost is a degree or description of clinical symptoms that correlates with a numerical range on the 0–3 scale.

##### Roundtable case review

We compiled all providers’ Type 2 PGA scores for the 30 cases. In a roundtable on 3 September 2020, we discussed 14 cases, starting with those with the most disparate scores. After reviewing these cases together and coming to a consensus about scoring, the providers then rescored the remaining cases. On 24 September 2020, the remaining cases were discussed in a second roundtable. Meetings were recorded, with notes taken.

#### Step 2: psychometric evaluation

Following the second roundtable, the Type 2 PGA was psychometrically evaluated using data collected from the DLR (from 25 September 2020 to 10 October 2021).^15^

##### Construct validity

The construct validity of the Type 2 PGA was determined by estimating the Spearman correlation coefficients between Type 2 PGA scores with the following patient-reported measures of type 2 symptoms: total PSD scores; areas of pain; SSS; PSD scores for fatigue severity, cognitive symptom severity and waking unrefreshed; and SLAQ scores for fatigue, forgetfulness, anxiety, muscle weakness and muscle pain. Additionally, we estimated the correlation between Type 2 PGA scores with physician-reported measures of type 1 SLE activity: SLEDAI, clinical SLEDAI (scored without labs) and Type 1 PGA. Correlations were defined as no correlation (r=0), weak (r=0.1–0.3), moderate (r=0.4–0.6), strong (r=0.7–0.9) and perfect (r=1.0).^16^ We hypothesised that there would be strong correlations between the Type 2 PGA and patient-reported measures of type 2 symptoms, indicating convergent concepts, and weak correlations between the Type 2 PGA and physician-reported measures of type 1 SLE activity, indicating divergent concepts. We estimated we would need to analyse 193 paired Type 2 PGA and PSD scores to have 80% power to detect a minimum correlation of 0.2.

##### Known groups validity

The known groups validity analysis determined whether the Type 2 PGA could differentiate the levels of severity of type 2 symptoms reported by patients. We compared the average Type 2 PGA score and 95% CI across the levels of PSD scores: none (0–3), mild (4–7), moderate (8–11), severe (12–19) and very severe (20–31).^3^ Differences in mean Type 2 PGA score across PSD severity categories were estimated by analysis of variance (ANOVA). Additionally, we hypothesised that there would be lower mean Type 2 PGA scores in patients with lower PSD severity. We therefore compared the average Type 2 PGA scores of patients with none to mild PSD scores (0–7) with the average Type 2 PGA scores of those with moderate to very severe scores (8–31). We estimated we would need 200 patients to have 80% power to detect a minimum anticipated effect size (Cohen’s *d*) of 0.4 comparing the average Type 2 PGA scores between PSD categories of none to mild (<8) and moderate to very severe (≥8).

##### Test–retest reliability

To assess test–retest reliability, the Type 2 PGA scores were compared between two time points for patients with *no change* in the patient-reported PSD, defined as a second PSD score within one point of the initial PSD score. A reliable measure would have consistent Type 2 PGA scores over time among patients without changes in type 2 symptoms. Reliability was estimated by an intraclass correlation coefficient (ICC), defined as poor (<0.5), moderate (0.5–0.75), good (0.75–0.9) and excellent (>0.90).^17^ We estimated we would need to analyse 50 patients with stable PSD and two Type 2 PGA scores at separate visits to have 90% power to detect a minimum ICC of 0.4.

##### Responsiveness to change

The responsiveness to change was determined by comparing the change in Type 2 PGA score with the change in the PSD score between visits. We hypothesised that as the PSD changed so would the Type 2 PGA. Patients who had two visits following the roundtable were included in the analysis. Responsiveness was determined by estimating the Spearman correlation coefficients between the change in Type 2 PGA scores and the change in PSD scores, using the criteria defined above.^16^ We estimated we would need to analyse 193 paired Type 2 PGA and PSD scores to have 80% power to detect a minimum correlation of 0.2.

##### Intra-rater and inter-rater reliability

Fifty patients with visits after the second roundtable were selected to evaluate intra-rater and inter-rater reliability; this number was selected to have 90% power to detect a minimum ICC of 0.4. Cases were selected to represent a range of Type 2 PGA scores, type 1 SLE activity and patient demographics. For these 50 cases, each case was rescored by the initial treating rheumatologist and a second rheumatologist. Scoring was performed by six rheumatologists.

###### Intra-rater reliability

The intra-rater reliability measured the agreement within the same rheumatologist. To assess intra-rater reliability, 3 months after a patient visit, the treating rheumatologist rescored the Type 2 PGA by reading their clinical note and reviewing patient-reported measures (but without seeing their original score or patient name). Intra-rater reliability was estimated by an ICC and defined as poor (<0.5), moderate (0.5–0.75), good (0.75–0.9) and excellent (>0.90).^17^

###### Inter-rater reliability

The inter-rater reliability measured the agreement *between* two independent rheumatologists. To assess inter-rater reliability, a second rheumatologist scored a subgroup of 50 patients in the DLR after reviewing the patient-completed questionnaire, the clinical note of the visit being scored and the clinical note from the prior visit. The Type 2 PGA was scored and compared with the treating rheumatologist’s score without the second rheumatologist seeing the original score. Reliability was estimated by ICC, as defined above.

All analyses were conducted in SAS V.9.4.

### Patient and public involvement

Patients and/or the public were not involved in the design, conduct, reporting or dissemination plans of this research.

## Results

### Roundtable discussion

Six rheumatologists (five female; two Asian, four white and zero Hispanic; median age of 41 years; and median time practising in the Duke Lupus Clinic of 5 years, range: 2.5–13) scored and discussed 30 cases (57% with a history of nephritis, median age 37.5 years). The median Type 2 PGA score from the treating rheumatologists was 0.88 (IQR: 0.25–1.50) and the median PSD score was 11 (IQR: 6–22).

#### Proposed guideposts for scoring the Type 2 PGA

**Figure 1:**
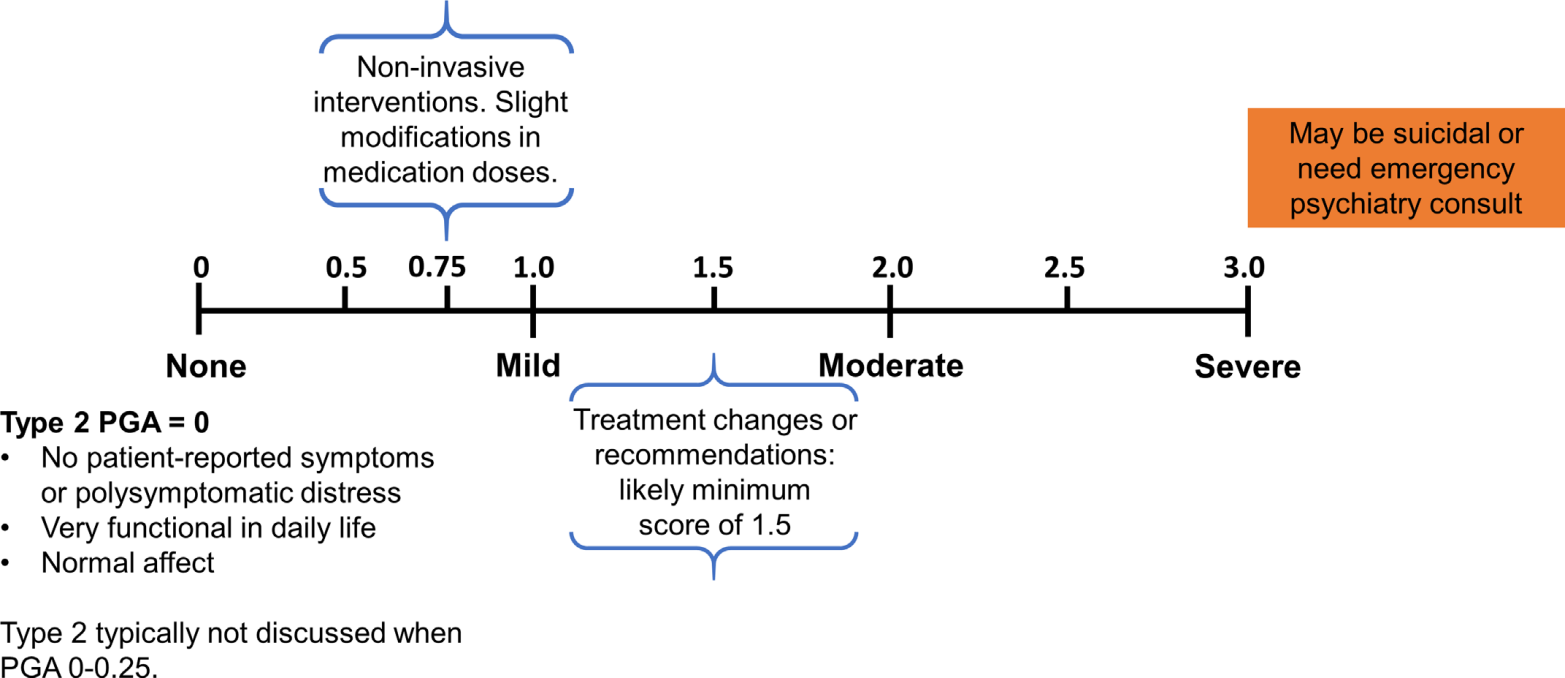
Agreed-on ‘guideposts’ for scoring the Type 2 PGA. PGA, Physician Global Assessment.

After discussing differences in scores across rheumatologists and what each rheumatologist considered when assigning a certain Type 2 PGA score, the group decided on clinical considerations to serve as ‘guideposts’ for the Type 2 PGA (figure 1).

- The provider’s impression of the impact of type 2 symptoms on a patient’s emotional, physical and social functioning drives the final Type 2 PGA score.
- Similar to the Type 1 PGA, 0 indicated no type 2 SLE activity, 1 indicated mild activity, 2 indicated moderate activity and 3 indicated severe activity.
- A Type 2 PGA score of 0 should only be assigned to a high-functioning patient with no type 2 SLE symptoms.
- A Type 2 PGA score of 1.5, similar to the Type 1 PGA, indicates an intention to escalate treatment or reinforce prior recommendations.
- A Type 2 PGA score of 3 should be assigned to a patient in severe distress, requiring high-level intervention or hospitalisation.
- Not all type 2 symptoms need to be experienced or be equally severe to have a high Type 2 PGA score.
- Symptom attribution can impact the Type 2 PGA. Non-lupus-related comorbidities or factors could lead a provider to decrease the Type 2 PGA score but would not take the score to 0.

The provider can derive an understanding of the patient’s symptoms and functioning through a combination of patient-reported surveys, history and/or physical exam. Examples of scoring are detailed in the following and additional examples of cases scored during the roundtable are presented in online supplemental table 1:

10.1136/lupus-2023-001016.supp1 Supplementary data

- In patients with avascular necrosis who report a PSD score of 15 because they have pain in multiple areas, cannot sleep and have depression due to the pain, we would score their Type 2 PGA between 1.0 and 2.0 depending on the symptoms’ impact on their quality life, physical function and clinical need for intervention.
- In patients with a PSD score of 4 with severe fatigue and ongoing depression without pain, but are tearful during the clinical encounter, are not able to attend work and are severely limited in their social and physical function, we would likely score their Type 2 PGA as 2.0–2.5.
- In patients with severe fatigue, brain fog and waking unrefreshed (PSD score of 9), but whose symptoms improve with sleep apnoea therapy, the Type 2 PGA score would likely range from 0.75 to 1.5. However, if the symptoms do not improve with sleep apnoea therapy, the Type 2 PGA score would range from 1.0 to 2.0.

### Psychometric evaluation

Following the roundtable and the development process for the Type 2 PGA, 263 patients had a visit in the Duke Lupus Clinic in which both Type 2 PGA and PSD scores were recorded. At the first visit following the roundtable, the median age of the patients was 44 years, 56% were black, 92% were female and 17% met the 2016 ACR criteria for fibromyalgia (table 1). The median Type 2 PGA and PSD scores were 0.50 (IQR: 0.25–1.50) and 8.0 (IQR: 4.0–14.0), respectively. The median clinical SLEDAI score was 0 (IQR: 0–2), and 10% had active nephritis.

**Table 1:** Duke Lupus Registry patient characteristics (N=263)

	n (%) or median (IQR)
Age, years	44 (33–54)
Black	148 (56)
Hispanic	13 (5)
Female	241 (92)
2016 ACR fibromyalgia criteria	44 (17)
Type 2 PGA	0.50 (0.25–1.50)
Polysymptomatic Distress Scale score	8.0 (4.0–14.0)
Type 1 PGA	0.50 (0–1.0)
Clinical SLEDAI	0 (0–2)
SLEDAI (n=234)	2 (0–4)
Active nephritis	22 (10)

ACR, American College of Rheumatology; PGA, Physician Global Assessment; SLEDAI, Systemic Lupus Erythematosus Disease Activity Index.

#### Construct validity

The Type 2 PGA was strongly correlated with the total PSD score and SSS and moderately correlated with the individual components from the PSD and SLAQ (table 2, online supplemental figure 1). Additionally, the Type 2 PGA was only weakly correlated with type 1 SLE measures of activity, indicating the Type 2 PGA measured a different construct.

**Table 2:** Spearman correlations of Type 2 PGA with patient-reported measures and type 1 SLE activity measures (N=263)

	r	P value
Total Polysymptomatic Distress Scale score	0.76	<0.001
Areas of widespread pain	0.59	<0.001
Symptom Severity Score	0.76	<0.001
Fatigue	0.68	<0.001
Cognitive dysfunction	0.63	<0.001
Waking unrefreshed	0.62	<0.001
SLAQ		
Forgetfulness	0.60	<0.001
Anxiety	0.55	<0.001
Fatigue	0.61	<0.001
Muscle weakness	0.62	<0.001
Muscle pain	0.56	<0.001
SLEDAI	0.18	<0.001
Clinical SLEDAI	0.32	<0.001
Type 1 PGA	0.34	<0.001

PGA, Physician Global Assessment; SLAQ, Systemic Lupus Activity Questionnaire; SLEDAI, Systemic Lupus Erythematosus Disease Activity Index.

#### Known groups validity

The mean Type 2 PGA score increased with each PSD category of severity, with non-overlapping 95% CIs between categories (table 3). Additionally, the mean Type 2 PGA score was higher in patients with PSD ≥8 (1.22, 95% CI 1.12 to 1.32) compared with PSD <8 (0.35, 95% CI 0.27 to 0.43, p<0.001).

**Table 3:** Average Type 2 PGA score across PSD score categories of severity (N=263)

PSD score	n	Type 2 PGA Mean (95% CI)	Type 2 PGA Median (IQR)	Type 2 PGA Range	P value
None: 0–3	54	0.19 (0.12 to 0.26)	0 (0–0.25)	0–1	<0.001
Mild: 4–7	68	0.47 (0.35 to 0.60)	0.25 (0.25–0.50)	0–2.5	
Moderate: 8–11	59	0.90 (0.78 to 1.02)	1.00 (0.50–1.25)	0–2	
Severe: 12–19	53	1.29 (1.13 to 1.46)	1.25 (0.75–1.75)	0.25–2.25	
Very severe: 20–31	29	1.72 (1.56 to 1.89)	1.75 (1.50–2.00)	0.75–2.50	
PSD <8	122	0.35 (0.27 to 0.43)	0.25 (0–0.50)	0–2.5	<0.001
PSD ≥8	141	1.22 (1.12 to 1.32)	1.25 (0.75–1.75)	0–2.5	

PGA, Physician Global Assessment; PSD, polysymptomatic distress.

#### Test–retest reliability

In a subset of 72 patients with a stable PSD score between two visits, the ICC between the Type 2 PGA scores at each visit was 0.69, indicating moderate reliability. The median time between visits was 119 days (IQR: 98–179), ranging from 12 to 245 days.

#### Responsiveness to change

**Figure 2:**
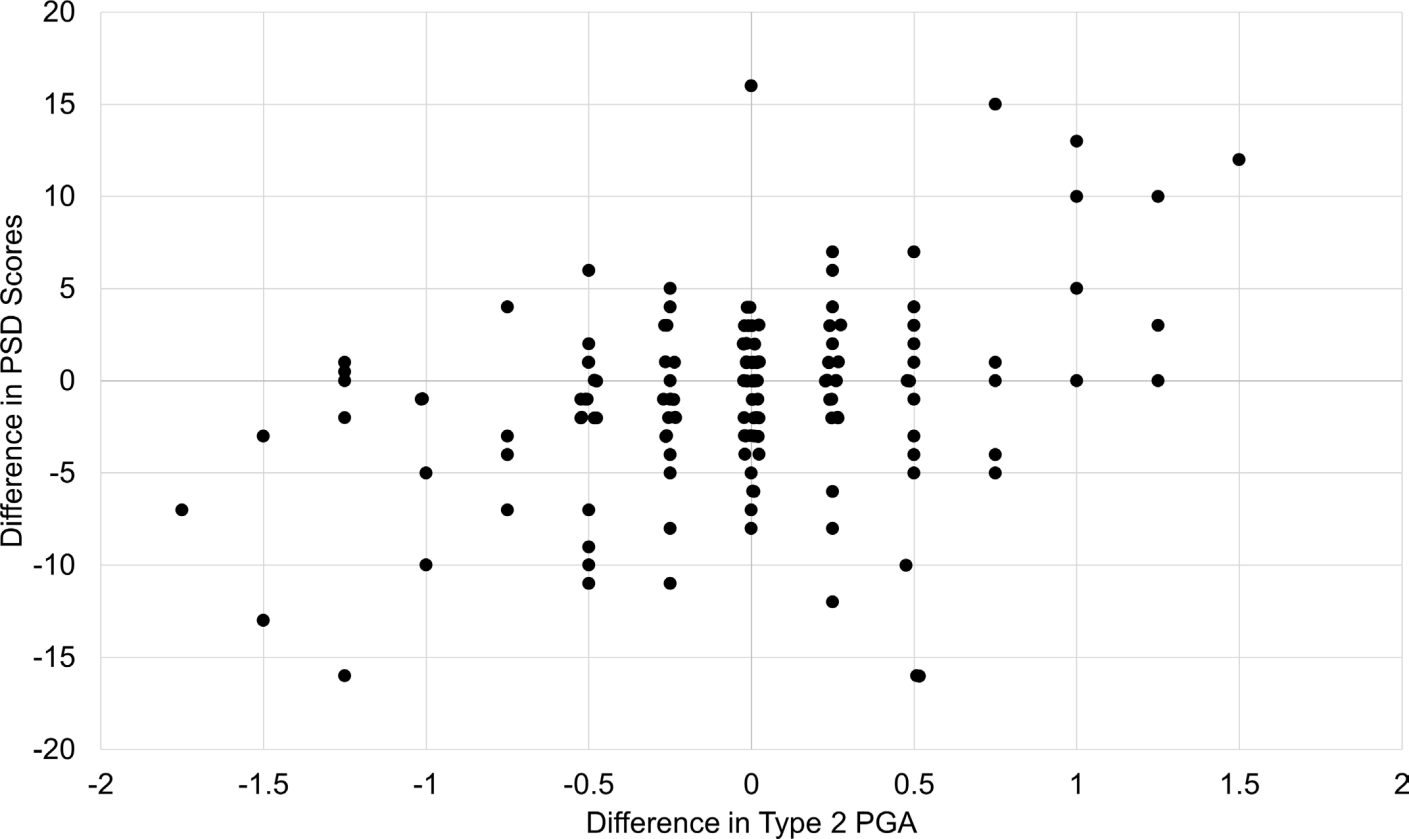
Responsiveness to change: correlation between change in Type 2 PGA scores and change in PSD scores between clinic visits (n=152; Spearman correlation: 0.28, p<0.001). When limited to patients who had at least a two-point difference in PSD scores between visits, the results were similar (Spearman correlation: 0.33, p=0.001). PGA, Physician Global Assessment PSD, Polysymptomatic Distress Scale.

In a subset of 152 patients with two visits, the median time between visits was 115.5 days (IQR: 91–165), ranging from 12 to 245 days. The median change in Type 2 PGA scores between visits was 0 (IQR: −0.25 to 0.25), and the median change between PSD scores was 0 (IQR: −3 to 1). There was a significant but weak correlation between change in Type 2 PGA scores and change in PSD scores between visits (r=0.28, p<0.001; figure 2). When limited to patients who had at least a two-point difference in PSD scores between visits, the results were similar (r=0.33, p=0.001).

#### Intra-rater and inter-rater reliability

Fifty cases were rescored to assess intra-rater and inter-rater reliability (70% black, 56% with a history of nephritis, median age of 43 years). The median Type 2 PGA score from the initial treating rheumatologist was 0.75 (IQR: 0.25–1.50), rescored by the treating rheumatologists was 0.75 (IQR: 0.25–1.25) and by the second rheumatologist was 0.75 (IQR: 0.50–1.25). The median PSD score was 7.5 (IQR: 4–13).

##### Intra-rater reliability

**Figure 3:**
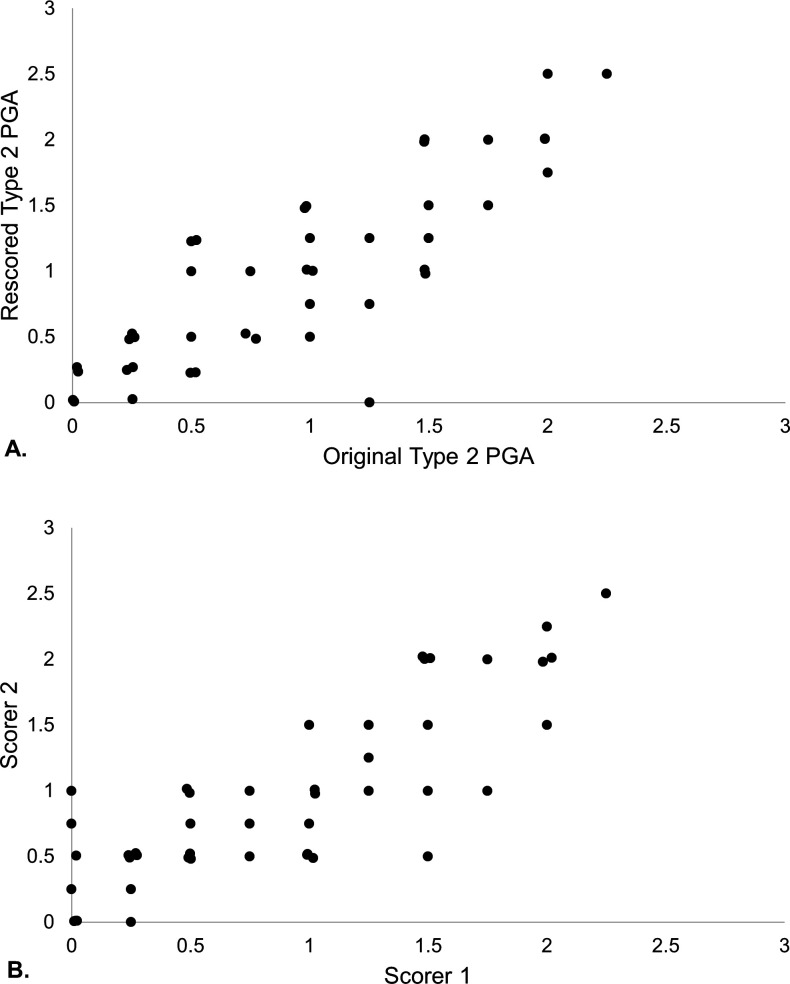
(A) Intra-rater reliability and (B) inter-rater reliability of Type 2 PGA (n=50). PGA, Physician Global Assessment.

When 50 cases were rescored by the same rheumatologist 3 months after the clinic visit, the median difference between the scores was 0 (IQR: −0.25 to 0.25). The ICC between the two scores was 0.83, indicating good intra-rater reliability (figure 3A).

##### Inter-rater reliability

When 50 cases were rescored by a different rheumatologist, the median difference between the scores was 0 (IQR: −0.25 to 0). The ICC between the two scores was 0.83, indicating good inter-rater reliability (figure 3B).

## Discussion

The Type 2 PGA provides a rheumatologist the opportunity to assess and quantify a range of symptoms through the lens of the Type 1 and 2 SLE Model. In this study, we found the Type 2 PGA to have reliable, reproducible and strong correlations with patient-reported symptoms of pain, fatigue and brain fog. The Type 2 PGA measure was designed to better assess a prominent group of symptoms that frequently and significantly impair the quality of life of patients with SLE, and yet are not considered evidence of disease activity using most conventional measures.^5 18–23^ These symptoms are, however, typically included in patient-reported measures in lupus.^10 24–26^ Traditionally, type 2 SLE symptoms may be viewed differently by patients and rheumatologists. While patients often consider these symptoms as manifestations of active SLE, rheumatologists may view them as comorbidities or as the non-specific consequences of living with a chronic disease. By developing the Type 2 PGA to complement the Type 1 PGA, this study provides the foundation for a more comprehensive approach to personalised care.

The Type 2 PGA yielded construct validity correlations that were consistent with our hypothesis that it would substantially correlate with the PSD and less so with measures of type 1 SLE. Further, the data indicate that each increase in PSD, from no symptoms to mild, moderate, severe and very severe, was accompanied by a stepwise increase in the Type 2 PGA by 0.3–0.5 points. Within this study, the PSD proved to be a useful measure of type 2 SLE. The PSD is not specific to SLE, and symptoms of pain, fatigue and cognitive dysfunction can be associated with many different conditions seen in patients with SLE, including fibromyalgia, acute infection, anasarca, arthritis, pregnancy, depression, insomnia, sleep apnoea, thyroid dysfunction, severe anaemia and many others. When symptoms result from another medical condition, the patient is likely to report a higher PSD than the provider attributes to type 2 SLE.

In this study, for some patients, the PSD score was lower than expected for the assigned Type 2 PGA score. This situation could occur in patients who experience one very severe type 2 SLE symptom, such as fatigue, that is not reflected in a high PSD score. Additionally, patients may under-report symptoms on the survey due to a number of reasons, including survey fatigue, adjustments in perceived norms for type 2 symptoms, efforts to hide these symptoms from a partner or provider, or reluctance with reporting these symptoms on a form. In this situation, type 2 symptoms were elicited by the provider through discussion and examination of the patient, as well as the provider’s assessment of the patient’s functionality, demonstrating a survey alone may not always be sufficient to underpin decisions on patient care.

In our experience, some patients with active type 1 SLE will report a high level of symptoms on the PSD. There can be concordance between the PSD and type 1 SLE in at least three clinical scenarios: (1) severe inflammatory arthritis leading to pain in multiple areas and poor sleep; (2) severe lupus nephritis with anasarca causing diffuse pain; or (3) an increase in PSD symptoms prior to or during a flare of type 1 SLE. While in the first two scenarios the PSD symptoms are likely secondary to the pain of specific type 1 manifestations, the third scenario suggests that some type 2 symptoms may be physiologically related to type 1 inflammation. Our previous qualitative work supports the idea that some patients with SLE only experience type 2 symptoms in association with their type 1 symptoms; we have defined this pattern as intermittent type 2 SLE.^27^

We observed high intra- and inter-rater reliability with minimal differences in scoring between providers, suggesting this score could be reliably used by other clinical providers. However, the alignment of the clinical team with PGA scoring based on a shared clinical practice and two roundtable discussions may limit the generalisability of our results. An additional limitation of our study is that all patients were seen at a tertiary care lupus clinic and may not be representative of all individuals living with SLE. Future studies should evaluate the psychometric properties of the Type 2 PGA at other institutions.

Accurately assessing the magnitude and origin of patient symptomatology is an essential element of effective patient care. The Type 1 and 2 SLE Model allows providers to interpret patient-reported measures in people living with SLE. By considering the common symptoms of pain, fatigue and brain fog together as type 2 SLE, the rheumatologist can assign a score to these frequent and debilitating symptoms. Incorporating the Type 2 PGA into routine rheumatology visits can improve patient care by bringing the provider’s attention to a specific group of symptoms not well represented in conventional measures of disease activity. Our first pilot study of the Type 2 PGA demonstrated the value of assessing and documenting the extent of type 2 symptoms by the rheumatologist. We found that implementing the Type 2 PGA increased efforts to manage type 2 symptoms. Management included the prescription of medications for type 2 symptoms, referrals to psychiatry, physical therapy, pain clinic and sleep studies, and counselling patients on lifestyle changes. With the Type 2 PGA, the number of interventions increased from 53% to 89% (p=0.03) for patients with high type 2 SLE activity.^13^

In conclusion, type 2 SLE symptoms can be reliably assessed and measured by rheumatologists using the Type 2 PGA. Through this simple, concise and reliable measure of type 2 symptoms, we believe we have taken an essential step towards enabling people living with SLE to have a meaningful reduction in symptoms and an improved quality of life.

## Data Availability

Data are available upon reasonable request.
