# Supplementary material for: Development and psychometric evaluation of a physician global assessment for type 2 systemic lupus erythematosus symptoms
Source: Lupus Sci Med. 2023 Dec 17;10(2):e001016. doi: 10.1136/lupus-2023-001016 (PMC10729218; doi:10.1136/lupus-2023-001016)
Supplement: Supplementary data [file lupus-2023-001016supp001.pdf]

Supplemental Table 1. Case examples of Type 2 PGA* scoring from the roundtable.

	Case 1	Case 2	Case 3
**Demographics**	68-year-old female, no LN† history, 15-20-year disease duration	44-year-old female, no LN history, 15-20-year disease duration	28-year-old female, LN history, 10-15-year disease duration
**Current Medications**	▪ aspirin 81 mg/day<br>▪ hydroxychloroquine 200 mg/day	▪ belimumab 200 mg subcutaneous/week<br>▪ hydroxychloroquine 300 mg/day<br>▪ methotrexate 25 mg/mL injection/week<br>▪ bupropion 150 mg XL tablet/day<br>▪ Latuda 60 mg tablet/day<br>▪ olanzapine 2.5 mg/day as needed<br>▪ buspirone 60 mg/day	▪ azathioprine 100 mg/day<br>▪ hydroxychloroquine 400 mg/day
**HPI‡**	Lupus is doing well. No arthritis, alopecia, rash, or ulcers. Some hip pain. Minimal fatigue.	Joints are aching and swelling, <30 minutes of morning stiffness. No energy. Wants to sleep all day. Slow to complete activities of daily living. Denies depression.	Severe burning rash on scalp and back. Unable to take lupus medications due to expense. Weight loss due to decreased appetite.
**Exam**	Trochanteric bursitis.	Tender MCPs§, wrist, elbows, right ankle, right foot. chronically ill appearing and appears fatigued. Numerous fibromyalgia tender points. Depressed affect.	Severe rash on scalp and body with open wounds and severe diffuse alopecia. Normal affect.
**SLEDAI¶**	0	0	6
**Type 1 PGA**	0	0.75	2.5
**PSD#**	5	28	4
**Type 2 PGA Roundtable Consensus**	0.25-0.5	2.25-2.75	0.25-0.5

*PGA= Physician's Global Assessment(5)

†LN= lupus nephritis

‡HPI= History of present illness

§MCPs= metacarpal phalangeal joints

¶SLEDAI = Systemic Lupus Erythematosus Disease Activity Index(1)

#PSD = polysymptomatic distress(2-4)

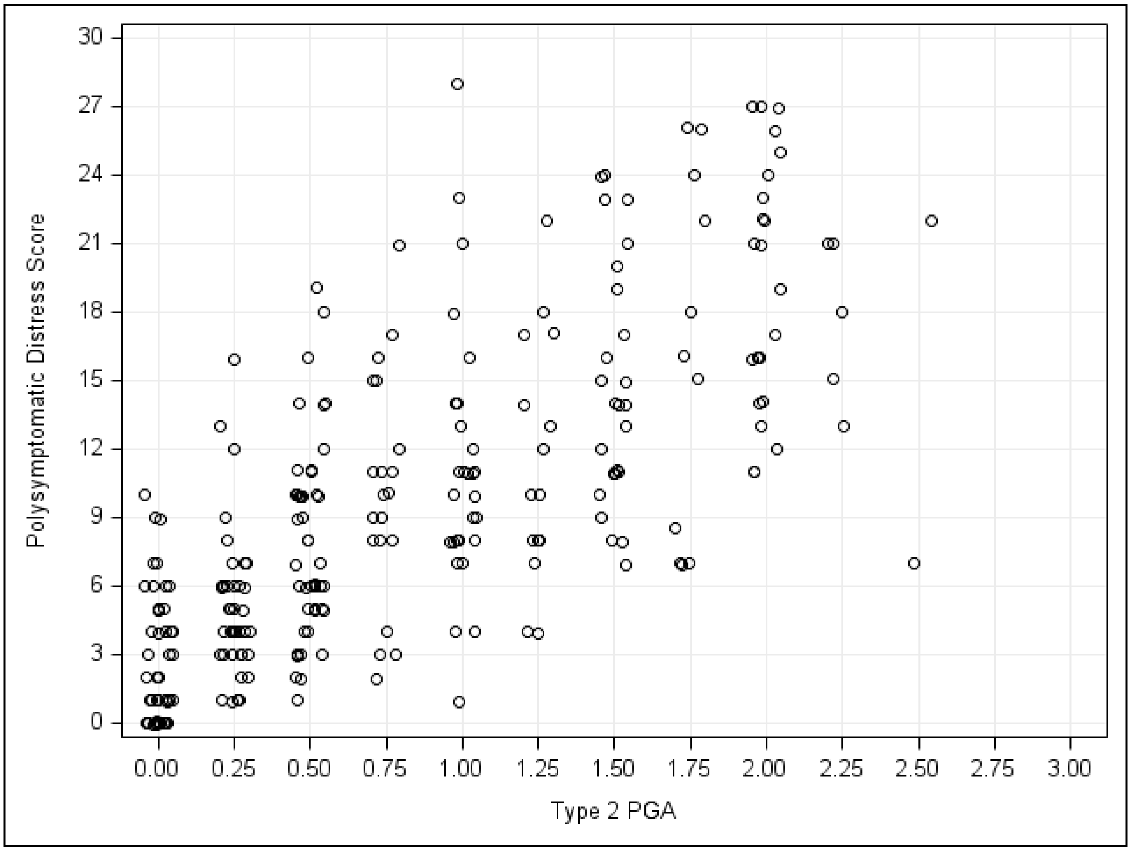

Supplemental Figure 1. Scatterplot of Type 2 PGA and Polysymptomatic Distress Score (rho=0.75; p<0.0001)
